# Self-reported efficacy of complementary and alternative medicine: the Akershus study of chronic headache

**DOI:** 10.1186/1129-2377-14-36

**Published:** 2013-04-18

**Authors:** Espen Saxhaug Kristoffersen, Kjersti Aaseth, Ragnhild Berling Grande, Christofer Lundqvist, Michael Bjørn Russell

**Affiliations:** 1 Head and Neck Research Group, Research Centre, Akershus University Hospital, Lørenskog, Norway; 2 Department of General Practice, Institute of Health and Society, University of Oslo, Oslo, Norway; 3 Institute of Clinical Medicine, Campus Akershus University Hospital, University of Oslo, Nordbyhagen, Norway; 4 Department of Neurology, Akershus University Hospital, Lørenskog, Norway; 5 HØKH, Research Centre, Akershus University Hospital, Lørenskog, Norway

**Keywords:** Complementary and alternative medicine, Primary chronic headache, Secondary chronic headache, Population-based, Chronic tension-type headache, Chronic migraine, Medication-overuse headache

## Abstract

**Background:**

Chronic headache is associated with disability and high utilisation of health care including complementary and alternative medicine (CAM).

**Findings:**

We investigated self-reported efficacy of CAM in people with chronic headache from the general population. Respondents with possible self-reported chronic headache were interviewed by physicians experienced in headache diagnostics. CAM queried included acupuncture, chiropractic, homeopathy, naprapathy, physiotherapy, psychological treatment, and psychomotor physiotherapy. Sixty-two % and 73% of those with primary and secondary chronic headache had used CAM.

Self-reported efficacy of CAM ranged from 0-43% without significant differences between gender, headache diagnoses, co-occurrence of migraine, medication use or physician contact.

**Conclusion:**

CAM is widely used, despite self-reported efficacy of different CAM modalities is modest in the management of chronic headache.

## Introduction

Chronic headache, i.e. ≥ 15 days/month for 3 months or ≥ 180 days/year affects 3-4% of the general population [1,2]. Management of chronic headache is a challenge, since medications often do not alleviate it sufficiently. Thus, many patients seek or are referred to complementary and alternative medicine (CAM), such as acupuncture, chiropractic, homeopathy, naprapathy, physiotherapy, psychological treatment and psychomotor physiotherapy. The use of CAM is high both in Norway and worldwide [3,4], and about 1 of 3 uses CAM for headache in Norway [5]. A survey among CAM providers suggests headache to be one of the conditions where patients benefit most from CAM management [6], but reports of treatment efficacy from the patients are lacking.

The aim of this study was to investigate self-reported efficacy of CAM in people with primary and secondary chronic headache from the general population.

## Findings

### Methods

A cross-sectional epidemiological survey, including 30 000 persons aged 30–44 years old stratified for age and gender, was drawn from the general population of eastern Akershus County, Norway. A short postal questionnaire screened for possible chronic headache (≥15days/last month and/or ≥180 days/last year). Screening-positive subjects were invited to a clinical interview and physical and neurological examination conducted by neurological residents. The criteria of the International Classification of Headache Disorders II (ICHD-II) were used with supplementary definitions for chronic rhinosinusitis and cervicogenic headache [1,2,7]. Chronic headache was defined as headache ≥ 15 days/months for at least 3 months or ≥ 180 days/year.

Medication overuse headache without other secondary causes was classified as primary chronic headache.

The Complementary and alternative medicine (CAM) forms queried included acupuncture, chiropractic, homeopathy, naprapathy (manipulation and stretching of joints and muscles), physiotherapy, psychological treatment and psychomotor physiotherapy.

All CAM use was included independently on whether it was reimbursed or not by the National Health Insurance. Homeopathy and naprapathy is not reimbursed, while the other CAM modalities, in some selected cases, are partially or fully reimbursed from authorised providers. The participants were asked for ever-use, e.g. “Have you ever tried/used/been to physiotherapy for headache?” For questions of self-reported efficacy, participants were asked with reference to the CAM management tried for their headache: “Did you experience any efficacy in terms of lasting reduction of headache frequency and/or intensity?”

A more detailed description of the material and methods has been given elsewhere [1,2,8].

#### Statistics

Statistical analyses were performed using SPSS 20.00 (SPSS Inc., Chicago, IL, USA). For descriptive data, proportions, means and 95% confidence intervals (CI) are given. Pearson χ^2^ test was used for testing significance of group differences for categorical data, Fisher`s exact test was used when appropriate. Significance levels were set at p<0.05. CI is not given when n <5.

#### Ethical issues

The Regional Committee for Medical Research Ethics and the Norwegian Social Science Data Services approved the study. All participants gave informed consent.

### Results

253/405 (62%) participants with primary chronic headache and 82/113 (73%) participants with secondary chronic headache had used CAM for headache. Table 1 shows the self-reported efficacy of CAM. Very few used homeopathy, naprapathy or psychological treatment.

**Table 1:** Self-reported efficacy of complementary and alternative medicine in people with primary and secondary chronic headache

	**CTTH without medication overuse % (95% CI) n/N**	**CTTH with medication overuse % (95% CI) n/N**	**CM % (95% CI) n/N**	**Other primary chronic headache % (95% CI) n/N**	**All primary chronic headache % (95% CI) n/N**	**CPTH % (95% CI) n/N**	**CEH % (95% CI) n/N**	**HACRs % (95% CI) n/N**	**Other secondary chronic headache % (95% CI) n/N**	**All secondary chronic headache % (95% CI) n/N**
***Acupuncture***	23 (15–35) (15/65)	16 (8–27) (9/58)	25 (7–59) (2/8)	0 (0–43) (0/5)	20 (14–27) (26/133)	32 (17–52) (8/25)	27 (10–57) (3/11)	41 (22–64) (7/17)	0 (0/1)	32 (20–46) (15/47)
***Chiropractic***	23 (14–34) (14/62)	28 (17–42) (13/47)	25 (1/4)	50 (1/2)	26 (19–34) (29/113)	30 (16–51) (7/23)	38 (14–69) (3/8)	43 (21–67) (6/14)	-	38 (24–53) (15/40)
***Homeopathy***	17 (1–37) (4/23)	18 (6–41) (3/17)	0 (0/2)	0 (0/3)	16 (8–29) (7/44)	13 (2–47) (1/8)	25 (1/4)	0 (0/7)	-	6 (1–28) (1/16)
***Naprapathy***	15(4–42) (2/13)	17 (3–56) (1/6)	-	0 (0/1)	15 (5–36) (3/20)	33 (1/3)	0 (0/1)	-	-	33 (1/3)
***Physiotherapy***	23 (17–32) (25/109)	28 (20–37) (26/94)	25 (7–59) (2/8)	25 (1/4)	25 (20–31) (53/211)	43 (29–58) (17/40)	28 (13–51) (5/18)	41 (23–61) (9/22)	33 (1/3)	38 (27–49) (27/72)
***Psychological treatment***	25 (1/4)	0 (0/3)	-	0 (0/1)	25 (7–59) (2/8)	0 (0/1)	-	33 (1/3)	-	33 (1/3)
***Psychomotor physiotherapy***	29 (12–55) (4/14)	41 (22–64) (7/17)	-	-	35 (21–53) (11/31)	50 (1/2)	0 (0/1)	0 (0/1)	-	25 (1/4)
***Any alternative treatment***	39 (31–47) (51/132)	42 (33–51) (46/110)	33 (12–65) (3/9)	33 (10–70) (2/6)	40 (34–46) (101/253)	51 (36–66)(21/41)	37 (19–59) (7/19)	50 (33–67) (15/30)	33 (1/3)	46 (36–57) (38/82)
***Mean number of CAM modalities used (range)***	2.3 (1–6)	2.3 (1–8)	2.6 (1–4)	3.2 (1–7)	2.4 (1–8)	2.7 (1–6)	2.5 (1–6)	2.4 (1–5)	1.3 (1–2)	2.5 (1–6)

*CTTH*; Chronic tension-type headache, *CM*; Chronic migraine, *CPTH*; Chronic post-traumatic headache, *CEH*; Cervicogenic headache, *HACRS*; Headache attributed to chronic rhinosinusitis.

Figures show percentages and numbers of patients who have report subjective efficacy of the given treatment (n) compared to the numbers who have tried the treatment (N). The diagnoses are not mutually exclusive, i.e. one person can have two or more headache diagnoses. CI are not given when N <5.

No significant differences were found in self-reported efficacy of different CAM modalities depending on gender, chronic headache diagnoses, co-occurrence of migraine, use of acute headache medication, use of prophylactic medication, medication overuse or physician contact.

In the subgroup of chronic tension-type headache (CTTH) without medication overuse acupuncture was reported to be effective in 38% of participants with co-occurrence of migraine compared to 11% of participants without such co-occurrence. (χ^2^, p=0.011).

## Discussion

### Methodological considerations

We asked the participants for ever use of different CAM modalities in headache management. The efficacy data are based on self-reports and therefore subject to recall bias. We are aware that personal causality, individual perception and understanding of pain in addition to belief in certain CAM modalities could affect the subjective measurement of headache relief and efficacy of CAM, i.e. placebo response. CAM efficacy was specified to the patients to entail a reduction in the chronic headache frequency and/or intensity. The number of data on homeopathy, naprapathy, and psychological treatment are low and results should be interpreted with caution. The term “complementary and alternative medicine” refers to a wide range of treatments that do not fall within conventional medicine. Definition of CAM varies, as the modalities included differs between therapeutic traditions, social and religious cultures, healthcare systems and legislations. Furthermore the field is constantly changing. This is also reflected in the US National Center for Complementary and Alternative Medicine definition of CAM as “a group of diverse medical and health care systems, practices, and products that are not presently considered to be part of conventional medicine” [9]. The prevalence of CAM use in the general population in Norway is lower than in Asia, US and Australia, but may be comparable to other north- and central European countries [3,4,10]. Treatment by a psychologist is in Norway generally not prescribed for headache except for patients who have a clear co-morbid illness within the psychiatric disorder spectrum. It is similar to the other CAM modalities here in that patients with few exceptions have to carry the all costs of this treatment themselves.

We have used the ICHD-II classification for headache diagnoses. The general aspects, limitations and strengths of this study have been discussed in details elsewhere [1,2,8].

### Results discussion

The use of CAM is high [11,12], and this indicates that people with chronic headache like other chronic pain sufferers are likely to use CAM as a treatment option [13,14].

Other studies have found that 40–90% of chronic headache sufferers in headache clinics use CAM for their headache, and that chronic headache sufferers are more likely to use CAM than episodic headache sufferers [15-17].

Although the use is high, the self-reported efficacy in our study is modest. This corresponds well with the results of two Italian studies [15,16]. Others have reported that medication-overuse headache patients perceive CAM treatment as ineffective compared to episodic migraine patients [15], but we did not find any difference depending on medication overuse or not, so the results from Italy might reflect a difference between chronic and episodic headache sufferers. The efficacy results of any CAM treatment and the number of CAM modalities used in our study indicate that the patients seek a 2nd or 3rd CAM treatment, if the previous modality failed or had insufficient efficacy.

The placebo response in the management of headache is approximately 30% in both pharmacological and non-pharmacological clinical trials [18,19]. The self-reported efficacy of CAM in our study is only slightly higher than the placebo effect. The efficacy of acupuncture was better in those with chronic tension-type headache (CTTH) and co-occurrence of migraine than in CTTH without co-occurrence of migraine. Otherwise we found no significant differences in the CAM efficacy. A recent Cochrane review of acupuncture for migraine prophylaxis [20] and a meta-analysis of manual therapies for migraine and cervicogenic headache shows it is likely to be as effective as prophylactic medication for migraine [21,22]. Thus, CAM might have an effect in some types of headaches.

Chronic headache is usually managed by medication, but medication may not always alleviate the condition, and some people do not tolerate acute and/or prophylactic medicine due to side effects or contraindications. Finally, as shown in other studies [15,16] some people may wish to avoid medication due to possible side effects or risk for medication overuse.

CAM might improve other health aspects than the chronic headache for which treatment is being sought. Thus, CAM may be a non-pharmacological alternative option in the management of headache for some people despite the contrast between the widespread use of CAM and the lack of robust scientific evidence for the efficacy of all these therapies. Randomised controlled trials (RCTs) of CAM in headache management are sparse and for certain modalities there are no RCTs published in the literature. This indicates that there is a high and unmet need for high-quality research in this field. New studies are needed and these should be methodologically robust and follow the clinical trial guidelines from IHS in order to provide data for the rationale of CAM management in headache.

## Conclusion

CAM is widely used, despite self-reported efficacy of different CAM modalities is modest in the management of chronic headache.

## Competing interest

All authors declare that they have no competing interests.

## Authors’ contributions

MBR had the original idea for the study and planned the overall design. ESK prepared the initial draft and was the main author of the present manuscript. RBG and KA collected data. CL and MBR was involved in data analysis and interpretation and assisted in preparation of the manuscript. All authors have read, revised and approved the final manuscript.
